# A Case Report of Life-Threatening Hemopneumothorax as a Result of Spinal Manipulation Performed by Chiropractor

**DOI:** 10.7759/cureus.18031

**Published:** 2021-09-16

**Authors:** Sohaib Khatib, Taher Sabobeh, Khalid Abdalla, Salil Kulkarni

**Affiliations:** 1 Internal Medicine, University of Missouri Kansas City School of Medicine, Kansas City, USA; 2 Pulmonary and Critical Care Medicine, University of Missouri Kansas City School of Medicine, Kansas City, USA

**Keywords:** spontaneous pneumothorax, tube thoracostomy, spinal manipulation, hemopneumothorax, chiropractic

## Abstract

Chiropractic is a very popular alternative medicine practice in the United States. Despite that, this practice has been associated with several complications raising concerns for its safety. We report the case of an otherwise healthy 36-years-old, tall and thin male who presented with sudden onset shortness of breath associated with chest pain two days after chiropractic spinal manipulation. Chest imaging revealed left-sided hemopneumothorax required treatment with left-sided chest tube placement. Patients with a high risk of developing primary or secondary pneumothorax should consider avoiding chiropractic chest or spinal manipulations due to possible complications.

## Introduction

The use of alternative medicine has dramatically increased over the last several decades with about 62.1% of Americans used one of the alternative medicine therapies in one year [1]. Chiropractic is one of the largest complementary and alternative medicine (CAM) professions in the United States [2]. Despite the increasing popularity of chiropractic practice, there are still concerns regarding the safety of this practice.

Hemopneumothorax involves the accumulation of blood and air in the pleural space. Most cases of hemopneumothorax occur secondary to chest trauma; however, few cases of spontaneous hemopneumothorax without evidence of trauma have been reported [3]. Here, we report a case of life-threatening hemopneumothorax that was precipitated by chest and back manipulation performed by a chiropractor.

## Case presentation

The patient reported in our case is a 36-years-old, tall and thin (height: 185.5 cm, weight: 70.3 kg, BMI: 20.4) male without a significant past medical history. Social history revealed a 30 pack-year cigarette smoking, the patient quit smoking five years before the presentation.

The patient reported left-sided back pain for three days. He went the next day after the pain started to a chiropractor and underwent spinal manipulation of his back to relieve his back pain. He described a significant improvement in his back pain after that. However, he suddenly developed shortness of breath at rest and left-sided chest pain a day later, and he presented to the emergency department for evaluation.

**Figure 1:**
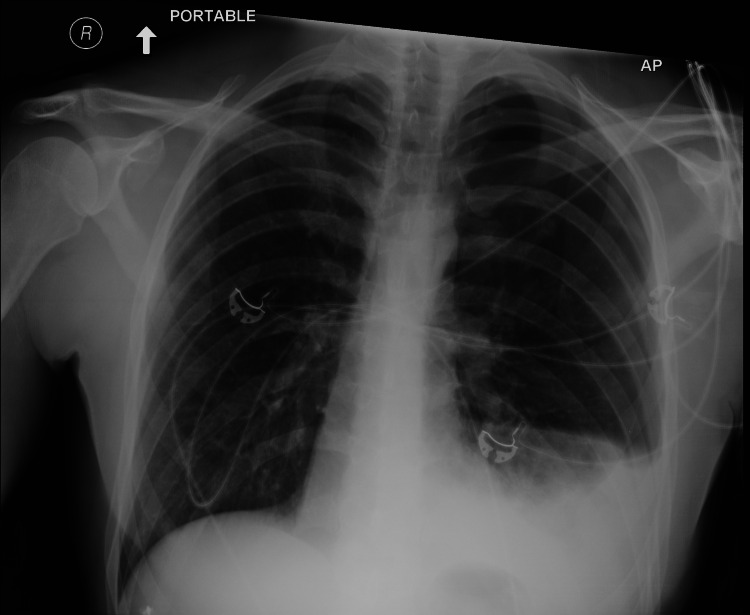
Chest x-ray. Chest x-ray showing a moderate left-sided hydropneumothorax. White arrow points toward the patient's head.

At presentation, the patient was afebrile, tachycardic with a heart rate of 105, normotensive with normal oxygen saturation (96% on room air). Chest x-ray showed a moderate left-sided hydropneumothorax (Figure 1).

**Figure 2:**
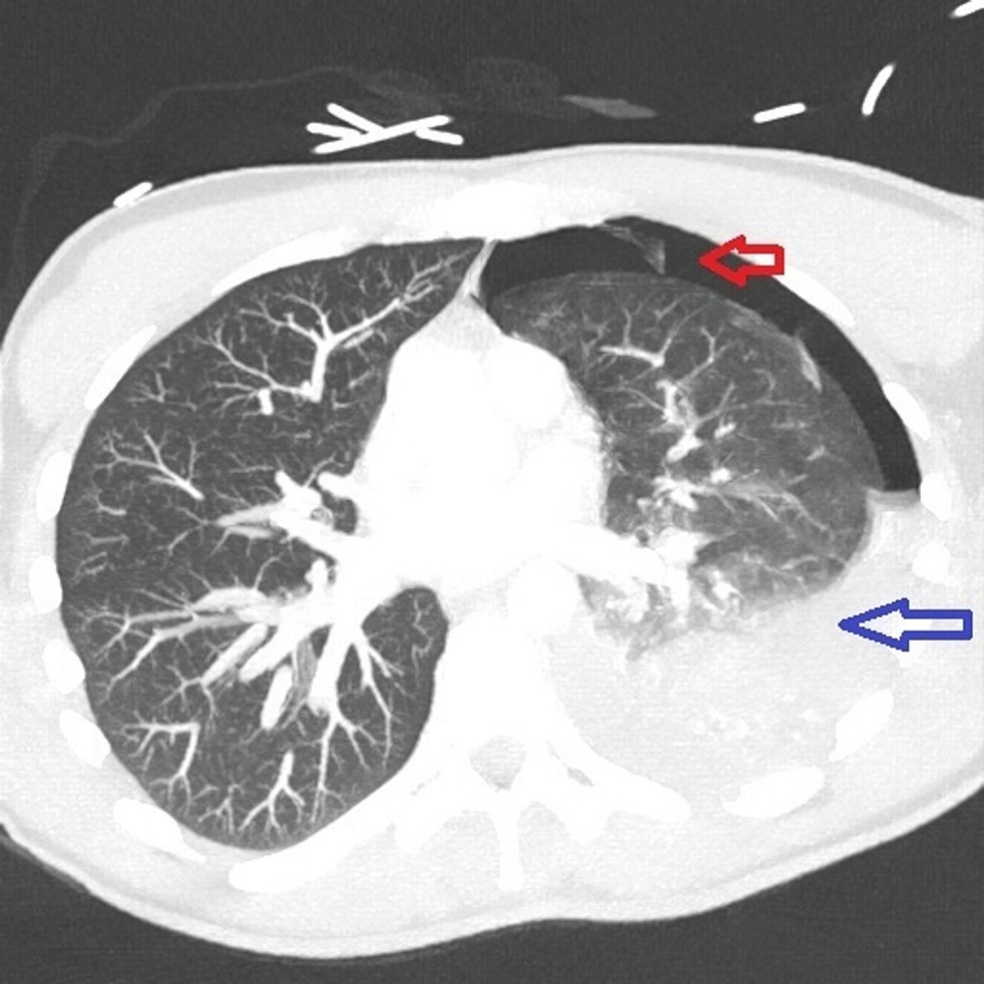
Chest CT scan with contrast. Chest CT scan with contrast showing moderate left-sided hydropneumothorax, red arrow points to pneumothorax component, and blue arrow points to hemothorax component.

**Figure 3:**
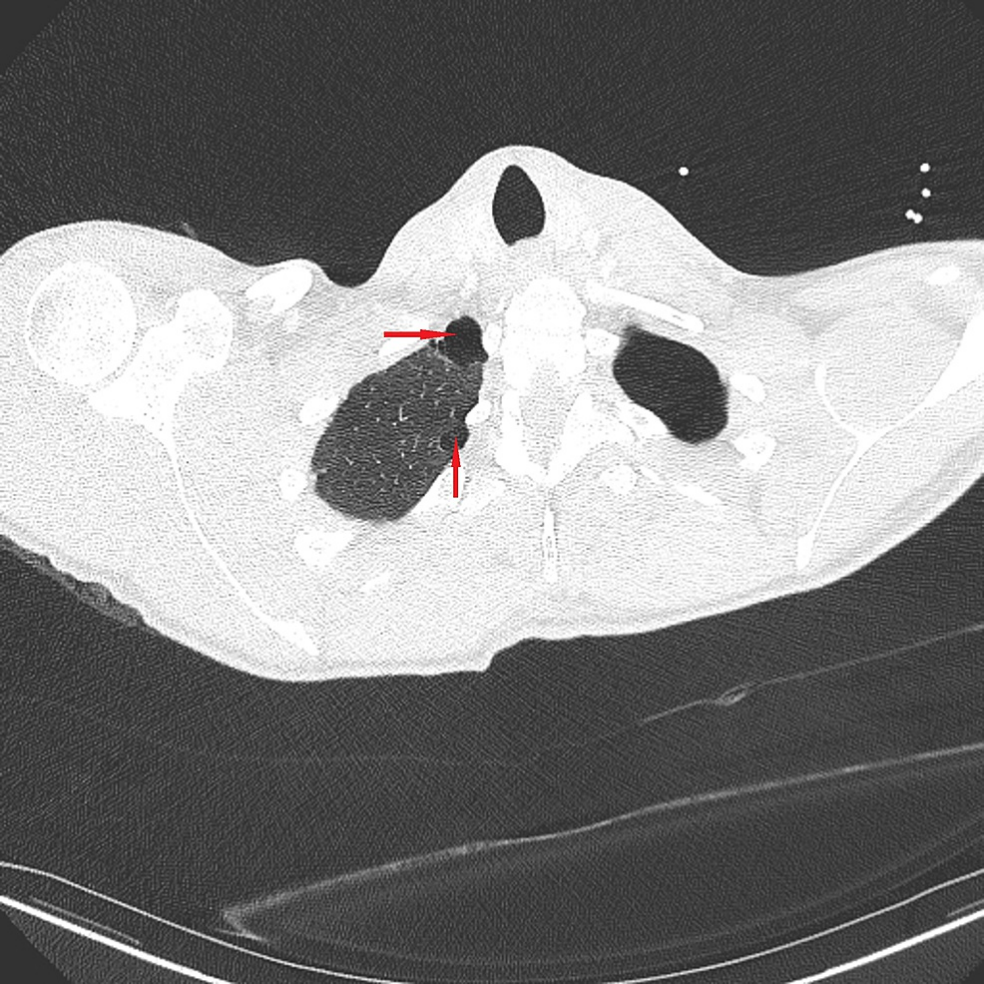
Chest CT scan with contrast. Chest CT scan with contrast showing mild right-sided apical paraseptal blebs, red arrows.

Chest CT scan with contrast was then done and showed moderate left-sided hydropneumothorax with moderate pneumothorax component and moderate pleural fluid component (Figure 2). It also showed mild right-sided apical paraseptal blebs (Figure 3).

**Figure 4:**
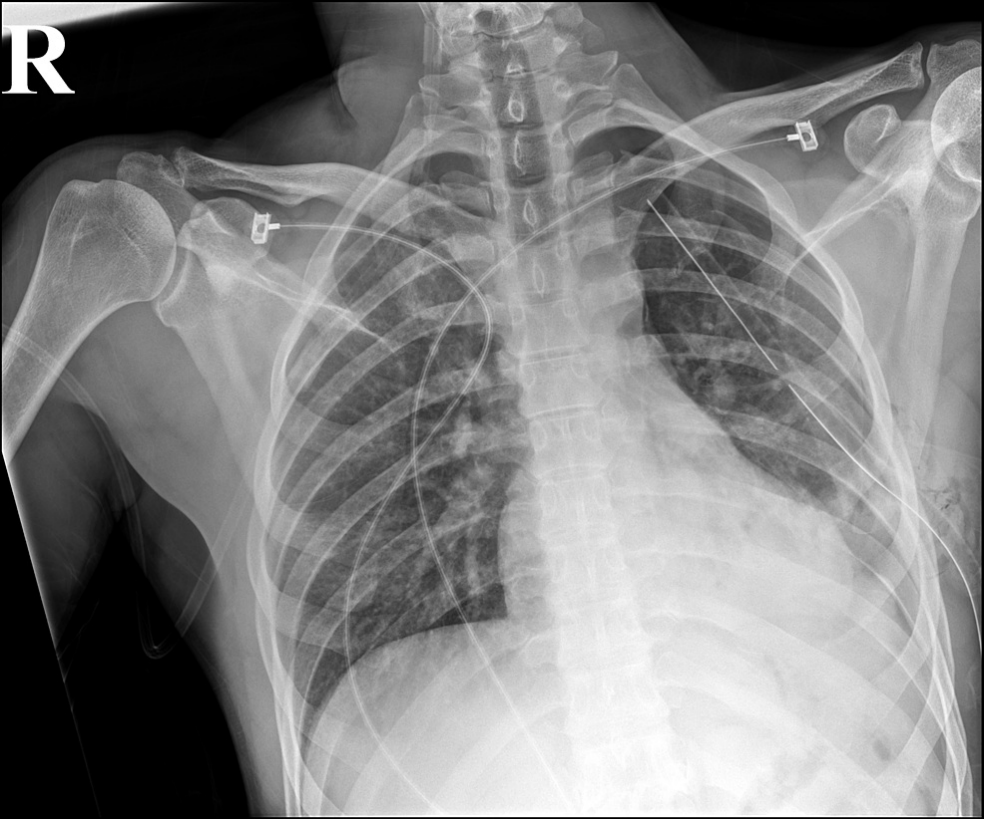
Chest x-ray after tube thoracostomy. Chest x-ray confirms good left-sided chest tube placement with improvement in left-sided hydropneumothorax.

After that, a left-sided chest tube was inserted with immediate drainage of 700 cc of bloody output. It was then placed on negative 20 mmHg suction. A chest x-ray confirmed a good chest tube placement with decreased left hydropneumothorax (Figure 4).

**Figure 5:**
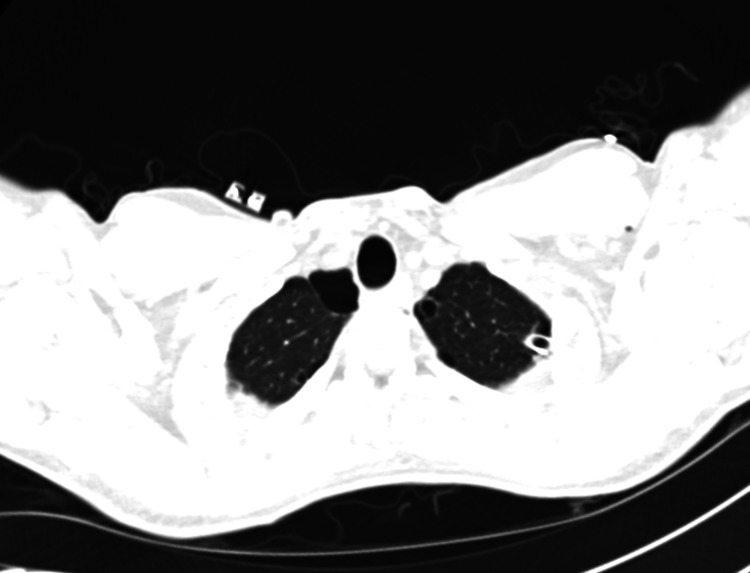
Chest CT angiogram. Chest CT angiogram negative for vascular aneurysms, dissection, or extravasation. It shows significant improvement in left hydropneumothorax with a small residual pneumothorax and significantly decreased residual pleural fluid.

The patient was then monitored with daily chest x-rays. Chest CT angiogram was performed on hospitalization day two that was negative for vascular aneurysms, dissection, or extravasation. It also showed significant improvement in left-sided hydropneumothorax with a small residual pneumothorax and significantly decreased residual pleural fluid (Figure 5).

On day two of hospitalization, chest tube drainage of 300 cc of the bloody component was noted. No further drainage on hospital day three. Therefore, the chest tube was clamped and then removed.

**Figure 6:**
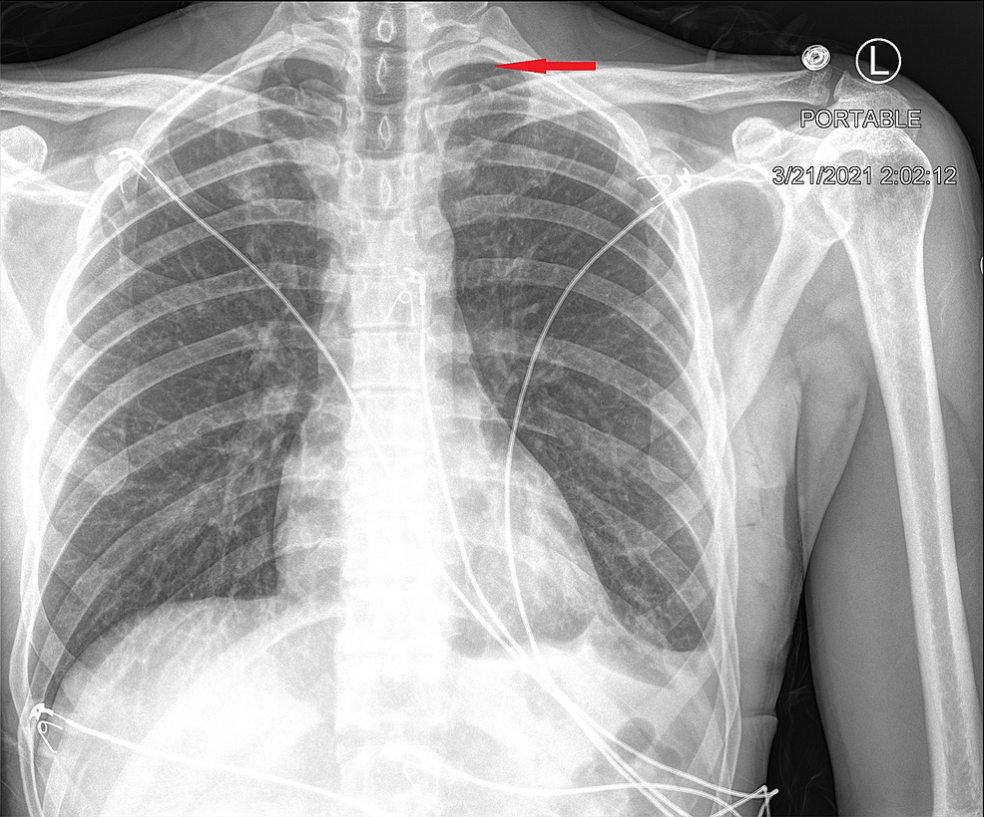
Chest x-ray on hospital day four. Chest x-ray on hospital day four showing new small left apical pneumothorax, the red arrow points to small left apical pneumothorax.

A morning chest x-ray on hospital day four showed a new small left apical pneumothorax, this was treated with high flow oxygen therapy (Figure 6). 

**Figure 7:**
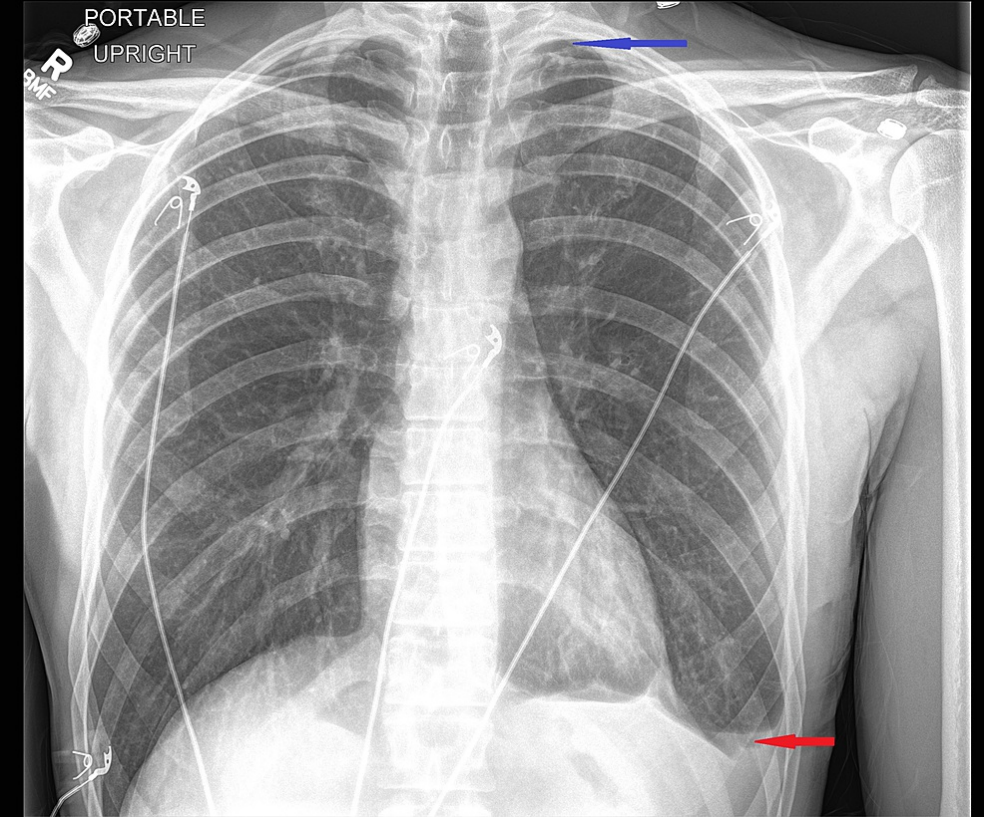
Chest x-ray on the day of discharge. Chest x-ray on the day of discharge showing stable left-sided pleural effusion (red arrow) and trace left-sided pneumothorax (blue arrow).

On the day of discharge, the patient was feeling well and denied any more shortness of breath or chest pain. The last chest x-ray on the day of discharge showed stable left-sided pleural effusion and trace left-sided pneumothorax (Figure 7).

**Figure 8:**
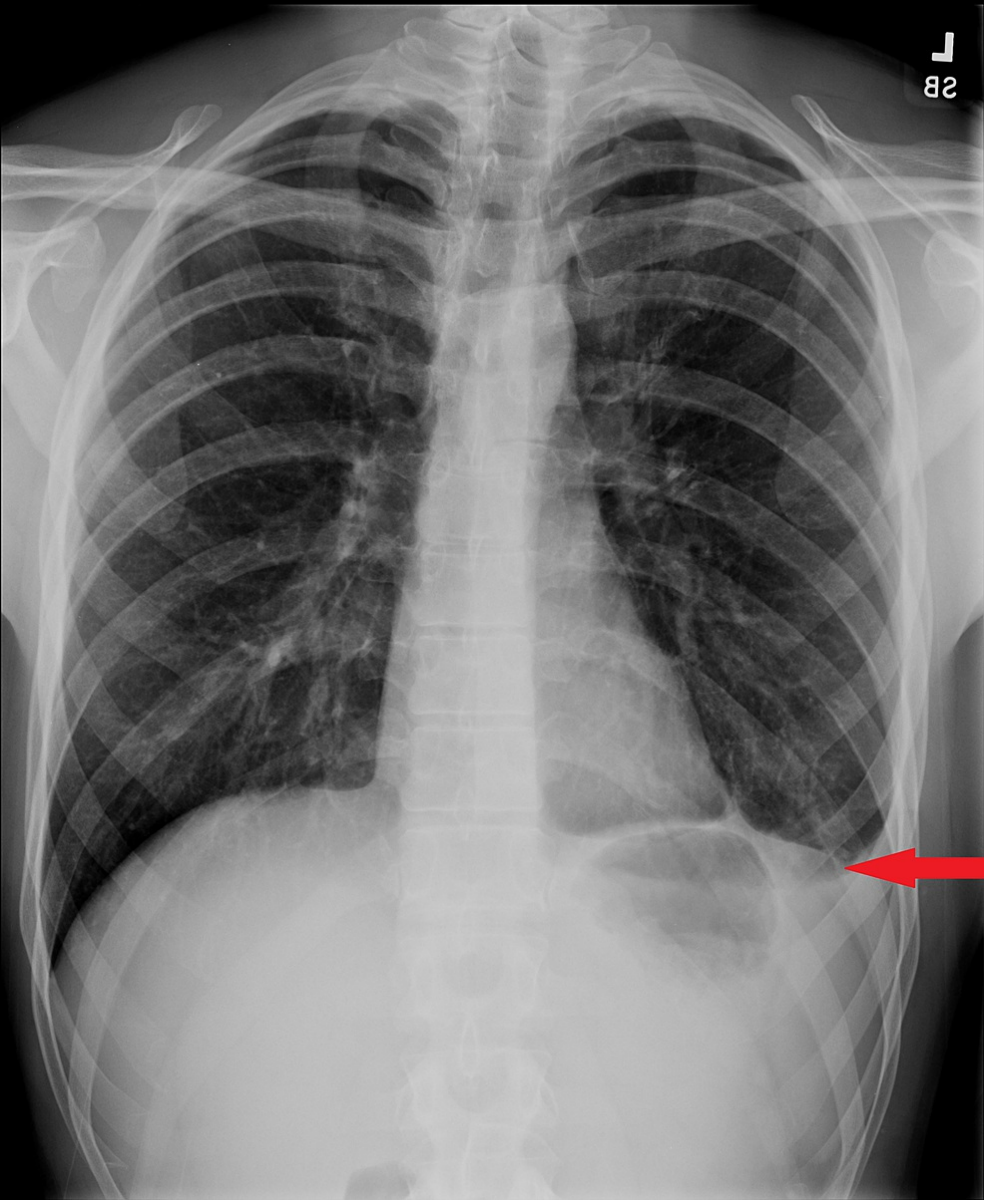
Chest x-ray done four days after discharge. Chest x-ray done four days after discharge showing only a persistent small left pleural effusion (red arrow) with no more left-sided pneumothorax.

Four days after discharge from the hospital, the patient followed with his primary care physician, who repeated a chest x-ray, and this showed only a persistent small left pleural effusion with no more left-sided pneumothorax (Figure 8).

## Discussion

Chiropractic is a health care profession recognized by World Health Organization (WHO), it focuses on the prevention, diagnosis, and treatment of several neuromusculoskeletal disorders. The two main types of therapeutic interventions in chiropractic practice include manipulation (high velocity, low amplitude thrusts that cannot be resisted by the patient) and mobilization (low-velocity passive motion that can be stopped by the patient) [4]. Despite the increasing popularity of chiropractic practice, there is no enough evidence to prove its effectiveness, in addition to that, there are increasing concerns about the safety of their interventions. Several neurological complications have been reported, such as arterial dissection, myelopathy, vertebral disc extrusion, and epidural hematoma [5,6]. 

Primary spontaneous pneumothorax is characterized by the spontaneous occurrence of pneumothorax in a patient without underlying lung disease. The most significant risk factor for primary spontaneous pneumothorax is smoking, which can cause decreased perfusion and impaired ventilation resulting in apical subpleural blebs [7]. It usually occurs in tall thin persons as pleural pressure is more negative at the apex of the lung. Spontaneous hemopneumothorax occurs in 1-12% of all cases with spontaneous pneumothorax. It likely occurs as a result of a torn adhesion between the parietal and visceral pleura or secondary to a rupture of vascularized bullae [3].

The patient in our case had significant risk factors for developing spontaneous pneumothorax as he had a significant smoking history with 30 pack-years. He also had the typical body habitus as a tall, thin person (height: 185.5 cm, weight: 70.3 kg, BMI: 20.4). CT scan of the chest also showed apical paraseptal blebs, which is a common finding seen in the imaging or pathological examination in patients with spontaneous pneumothorax [8]. We considered the occurrence of hemopneumothorax in our case as spontaneous and not traumatic as there were no manifestations of trauma on physical examination (such as bruises or hematomas) or imaging (such as rib fractures), besides, there was a time lag between the time of spinal manipulation and the onset of pneumothorax symptoms. We think that the repeated chest compressions from the spinal manipulation could have increased intrathoracic pressure and contributed to the rupture of lung blebs which ended in the development of pneumothorax. The hemothorax component might be explained by a torn adhesion between the parietal and visceral pleura or rupture of vascularized bullae.

Pulmonary complications associated with chiropractic interventions were previously reported in the literature. In 2007, Masneri et al. [9] reported a case of a 20-year-old woman who developed pneumothorax after attempted spinal manipulation by a layperson. Another case of a 17-year-old male who developed large and life-threatening hemothorax as a result of spinal manipulation was reported in 2013 [10]. To our knowledge, the presented case here is the first description of a life-threatening hemopneumothorax as a result of spinal manipulation.

## Conclusions

Chiropractor practice is a highly popular field; however, the safety of this field is still unclear. Patients with a high risk of developing primary or secondary pneumothorax should consider avoiding chiropractic chest and back manual interventions due to possible complications such as hemothorax, pneumothorax, and hemopneumothorax. 
